# Efficacy of MAP0004 in treating severe migraine

**DOI:** 10.1186/1129-2377-1-S14-P208

**Published:** 2013-02-21

**Authors:** S Kori, E Connors, J Zhou, B Lu, S Borland

**Affiliations:** 1 MAP Pharmaceuticals, USA

## 

The treatment needs of migraine patients are often unmet by available therapies, due in part to the incapability to completely relieve symptoms consistently across a broad spectrum of migraine attacks. MAP0004, an investigational drug that delivers dihydroergotamine (DHE) systemically via oral inhalation, was superior to placebo for the acute treatment of migraine in a Phase 3 trial. A subgroup analysis of subjects with severe migraine pain at baseline during the double blind period is reported here. Severe baseline pain was reported in 366 of the 794 subjects. Subjects with severe migraine pain treated with MAP0004 experienced statistically significant pain relief (p<0.05) as early as 10 minutes and at all subsequent pre-scheduled evaluation time points compared to placebo. These subjects were significantly pain free (p<0.05) by 60 minutes and at all subsequent time points following treatment compared to placebo. Sustained pain relief and sustained pain free values, both between 2 and 24 hours and 2 and 48 hours, were statistically significantly higher for MAP0004 relative to placebo. Headache recurrence over 24 hours occurred in 6.2% of severe migraine subjects treated with MAP0004 compared to 18% when treated with placebo. In summary, this analysis describes the baseline presentation of the severe migraine patient population and shows that MAP0004 was effective in the acute treatment of severe migraine in this Phase 3 trial.
